# EVALUATION OF GRA6 AS GENETIC MARKER FOR DETERMINING *TOXOPLASMA GONDII* GENOTYPE IN THE CEREBROSPINAL FLUID OF HIV/AIDS PATIENTS WITH TOXOPLASMIC ENCEPHALITIS

**DOI:** 10.21010/Ajidv19i1.4

**Published:** 2024-10-25

**Authors:** HARMINARTI Nora, SARI Ika Puspa, ARTAMA Wayan Tunas, IMRAN Darma, KURNIAWAN Agnes

**Affiliations:** 1 Doctoral Program in Biomedical Sciences, Faculty of Medicine Universitas Indonesia, Jakarta, Indonesia; 2 Department of Parasitology, Faculty of Medicine Universitas Andalas, Padang, Indonesia; 3 Department of Parasitology, Faculty of Medicine Universitas Indonesia, Jakarta, Indonesia; 4 Research Center for Biotechnology, Graduate school, Universitas Gadjah Mada; 5 Department of Neurology, Faculty of Medicine Universitas Indonesia-Cipto Mangunkusumo Hospital, Jakarta, Indonesia

**Keywords:** GRA6, genetic marker, genotyping, cerebrospinal fluid, Toxoplasmic encephalitis, PCR

## Abstract

**Background:**

Toxoplasmic encephalitis is a severe manifestation of *Toxoplasma gondii* infection, with potentially fatal outcomes, particularly among immunocompromised patients. Clinical manifestation of this infection is associated with a specific genotype of *T. gondii*, requiring the use of genetic marker for genotype determination.

**Aims:**

This study critically evaluated the application of GRA6 gene as genetic marker for genotyping *T. gondii* in cerebrospinal fluid (CSF) samples from HIV/AIDS patients diagnosed with Toxoplasmic encephalitis.

**Methods:**

The study analyzed 69 CSF samples from HIV/AIDS patients with Toxoplasmic encephalitis. These samples tested positive for *Toxoplasma* IgG serology and SAG2 PCR, while GRA6 genotyping was conducted using PCR-sequencing methods.

**Results:**

The results showed that GRA6 had potential for genotyping in positive control settings from culture cells. However, there was limited effectiveness in CSF samples from Toxoplasmic encephalitis patients.

**Conclusion:**

GRA6 had been proven effective as a genetic marker for the identification of *T. gondii* genotype among HIV/AIDS patients with Toxoplasmic encephalitis. However, the evaluation of GRA6 showed more effectiveness in cultured cells compared to direct clinical samples, such as cerebrospinal fluid obtained from HIV/AIDS patients with Toxoplasmic encephalitis.

## Introduction

Toxoplasmosis is a disease caused by the parasite *Toxoplasma gondii*, posing a significant risk for people with immunocompromise or foetuses. The encephalitic form of this infection is capable of affecting central nervous system and can be fatal for HIV/AIDS patients (Montoya and Liesenfeld, 2004). In Indonesia, cerebral toxoplasmosis is the most common form among HIV/AIDS patients (41%) (Maharani *et al.*, 2024), with clinical manifestations related to a specific genotype of *T. gondii*, which requires genetic marker for determination. (Xia *et al.*, 2017)

Several types of genetic markers have been used to detect *T. gondii*, with the dense granule group of protein GRA6 showing higher effectiveness. This protein is highly conserved and expressed throughout the parasite’s life cycle, serving as an ideal candidate for detecting *T. gondii* in clinical samples, including cerebrospinal fluid (CSF) from HIV/AIDS patients with Toxoplasmic encephalitis. GRA6 gene marker has enough polymorphism to detect three types of *T. gondii* genotype in various hosts. The investigation into the effectiveness of this genetic marker for the detection of *T. gondii* in Toxoplasmic encephalitis patients and genotyping presents a promising avenue of recent studies (Abedian *et al.*, 2024). By using GRA6 as a potential tool, there is possibility of increasing the accuracy and efficiency of diagnostic methods for HIV/AIDS patients as well as improving their treatment outcomes. (Vijaykumar *et al.*, 2018)

An advantage of using GRA6 as a diagnostic marker has been attributed to its high sensitivity. Several studies have shown that Polymerase Chain Reaction (PCR) assays using GRA6 can detect *T. gondii* DNA in clinical samples more effectively than other diagnostic methods. Therefore, the marker offers a promising option for diagnosis Toxoplasmic encephalitis, particularly in HIV/AIDS patients. The resulting PCR product is also applicable for genotyping. (Liu *et al.*, 2015; Khan and Noordin, 2020)

GRA6 has been widely used as marker for genotyping *T. gondii* due to its polymorphic nature, which allows the identification of different strains. However, the use of GRA6 is associated with limitations such as low levels of expression in certain strains of *T. gondii*, leading to negative results. The presence of inhibitors in clinical samples can affect the accuracy of PCR assays (Suwancharoen *et al.*, 2022). Despite numerous studies showing the potential of GRA6 as marker in *T. gondii* genotyping, there have been instances where GRA6-based genotyping has not been consistent or accurate using clinical samples. (Kurniawan *et al.*, 2020)

Despite the high prevalence of Toxoplasmic encephalitis in Indonesia, there is hardly any report regarding *T. gondii* genotype from clinical samples. The lack of reports shows the need to determine the variability of *T. gondii* genotype that causes Toxoplasmic encephalitis using genetic marker such as GRA6. Therefore, this study aimed to evaluate the use of GRA6 as a genetic marker for determining *T. gondii* genotype in direct clinical samples of HIV/ AIDS patients with Toxoplasmic encephalitis.

## Materials and Methods

### Sample Collection

CSF samples were collected from 69 HIV/AIDS patients with Toxoplasmic encephalitis referred by the neurologist at Cipto Mangunkusumo Hospital (RSCM), Jakarta, Indonesia. These samples were collected from patients with cerebral disorders diagnosed between 2013 and 2022. CSF samples were stored frozen at -70^0^C before DNA isolation. The inclusion criteria were patients with positive results for both Toxoplasma IgG serology ELISA (EUROIMMUN™, Lubeck, Germany) and SAG2 PCR.

### Ethical Approval

This study was approved by the University of Indonesia Ethical Committee under the reference number KET-770/UN2. F1/ETIK/PPM.00.02/2022 on August 1^st^, 2022.

### DNA isolation

CSF was preceded by centrifugation of 1 mL of samples for 10 minutes at 10.000 rpm, leaving 150 µL of sediment for boiling. Subsequently, DNA extraction was carried out by boiling the samples for 10 minutes at 100^0^C in dry bath. (Alfonso *et al.*, 2008)

### PCR Amplification

In this study, the detection of *T. gondii* was carried out through PCR. Initially, *T. gondii* infections were confirmed by nested PCR amplification targeting the repetitive and conserved gene SAG2. ((Mayashinta *et al.*, 2018; Halleyantoro *et al.*, 2019)

### Granule antigen 6 (GRA6) genotyping of *T. gondii*

PCR amplification was carried out in this study targeting GRA6 genetic marker. Primer sequences specific to GRA6 regions were applied to amplify the respective gene fragments examined for nested PCR using the Biorad C1000 Thermal Cycler, as shown in Table 1. Positive control samples used *T. gondii* RH strain, obtained from the Indonesian Research Centre for Veterinary Science. Specifically, positive control samples consisting of *T. gondii* tachyzoite isolates derived from in vitro culture, while the negative control template used Nuclease Free Water (NFW).

**Table 1:** Nucleotide sequences of PCR primers GRA6 used in this study

Genetic marker	Forward	Reverse	Amplicon (bp)	Reff
GRA6 Unnested	5’-GTAGCGTGCTTGTTGGCGAC-3’	5’ TACAAGACATAGAGTGCCCC-3’	791	(Fazaeli *et al.* 2000)(Vilares *et al.* 2017) (Zakimi *et al.* 2006)
GRA6 Nested	5’-TGT GGT GTT GGC AGT ATC TGT-3’	5’-CCCCTGTTTTCATCTTTAATA ATC-3’	240	(Gohari, Dalimi, and Pirestani 2020)

PCR master mix was prepared in a 20 µl volume reaction, comprising 10 IU of TopTaq® Master Mix (Qiagen, Germany), 2 µl of DNA template, and 0.2/0.4 μl of each primer. The concentration of MgCl2 was 0.8/0.4 mM for unnested/nested, respectively. For unnested PCR targeting GRA6, the optimum conditions included an initial denaturation step at 95°C for 5 minutes, followed by denaturation at 94°C for 30 seconds, annealing at 63.7°C for 1 minute, and extension at 72°C for 2 minutes. This cycle was repeated 40 times and concluded by extending the temperature to 72°C for 7 minutes. (Fazaeli *et al*. 2000) (Vilares *et al*. 2017)(Zakimi *et al*. 2006) For the nested PCR condition, the process started with an initial denaturation step at 95°C for 10 minutes, followed by denaturation at 94°C for 1 minute, annealing at 55°C for 30 seconds, and extension at 72°C for 1 minute. This cycle was also repeated 40 times and concluded by extending the temperature to 72°C for 7 minutes. (Gohari *et al.*, 2020)

### PCR Electrophoresis

Amplification result was separated on 2% agarose gel electrophoresis in 0.5x TBE buffer and stained with GelRed^®^ Nucleic Acid gel stain (Biotium, San Fransisco, USA) at a concentration of 1/10,000. Subsequently, electrophoresis was conducted at 100 volts for 40 minutes, and the result was observed under a 312 nm Vilber Lourmat™ UV Lamp, followed by the appearance of GRA6 as 240 bp bands.

### PCR Sequencing and Genotyping

In this study, PCR products were purified and subjected to Sanger sequencing. Subsequently, the sequences obtained were aligned with GRA6 reference sequence from the gene bank, using MEGA XI bioinformatics tools. Genotyping was conducted by comparing the obtained sequences with known *T. gondii* genotype (Tamura, Stecher, and Kumar 2021). The sequences of GRA6 locus were arranged with Type I — RH (accession no: JN649063, AF239283, AB235433), Type II — ME49 (accession no: JX044213, MT321285), and Type III — NED (accession no: AF239286, AF239288)

## Results

**Figure 1:**
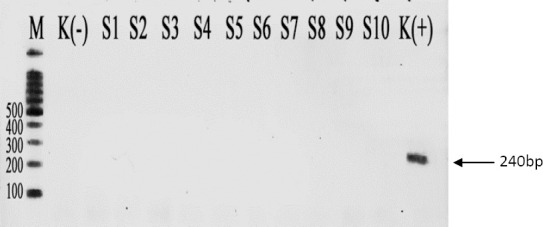
GRA6 polymerase chain reaction products of some positive SAG2 PCR. Lane M: 100 bp DNA ladder; K(-) ; Negative control; S1-S10; samples; K(+): Positive control.

**Figure 2:**
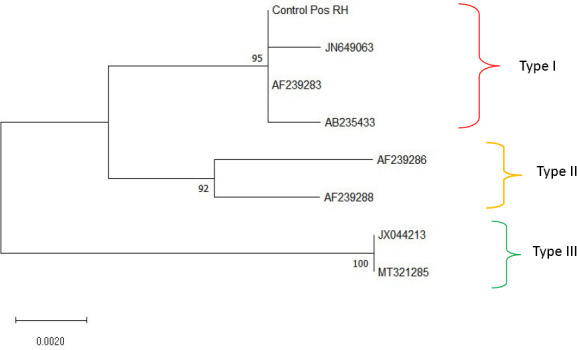
Phylogenetic tree analysis of GRA6 locus from the positive control *T. gondii* RH strain showing type I. *T. gondii* type I-III reference sequences were retrieved from the genebank. Phylogenetic tree was generated with 1000 bootstrap replicates using the Maximum Likelihood method and Tamura-Nei model.

An extensive analysis of CSF samples from patients with HIV/AIDS and Toxoplasmic encephalitis was carried out to determine *T. gondii* genotype. GRA6 PCR that targeted all 69 CSF samples showed negative results, subsequently sequencing and genotyping were not carried out. However, positive control of RH strain *T. gondii* tachyzoite showed positive result (Figure 1), followed by sequencing and phylogenetic analysis. The phylogenetic tree resulted as type I (Figure 2). These results provided valuable information, supporting previous studies on the inefficacy of GRA6 as genetic marker when performed directly on the clinical samples.

## Discussion

This study aimed to evaluate the use of GRA6 as genetic marker for the determination of *T*. *gondii* genotype in direct clinical samples of HIV/AIDS patients with Toxoplasmic encephalitis. The evaluation was carried out due to the absence of reports or data on *T. gondii* in patients with Toxoplasmic encephalitis and genetic marker used in Indonesia.

The target of the study is GRA6 gene, which is capable of distinguishing between the three types of *T. gondii*, playing a role in encoding a solid granule protein crucial for parasite antigenicity and pathogenicity. Due to the diverse genetic variability, GRA6 serves as an important marker for characterization and genotype determination. This marker also has a sequence with a high degree of polymorphism, correlating with the phenotype of the *T. gondii* strain. Furthermore, it can encode approximately 230 amino acid polypeptides, with the difference in each type of *T. gondii*, showing potential in antigenicity and pathogenicity. Various amino acid changes in GRA6 protein are related to virulence differences between genotype I and II (Nam 2009). During host cell infiltration, *T. gondii* releases a protein called GRA6 into the parasitophorous vacuole (PV), facilitating survival in the intracellular environment by traversing the PV membrane. Although the precise function of GRA6 in the *T. gondii* life cycle is not fully understood, there is interaction with host proteins and regulated responses (Nam 2009; Zhang *et al.*, 2019).

In this study, the analysis was carried out using GRA6 to identify positive *T. gondii* samples from previous tests through *Toxoplasma* IgG serological ELISA and PCR SAG2. However, during PCR examination with GRA6, negative results were found on all samples. These results showed distinctive variation compared to a successful study conducted in Brazil that reported positive outcomes in CSF, where the parasite in Vero cells was cultured before genotyping (Ferreira *et al.*, 2008; Bastos da Silva *et al*. 2016). Although the same positive controls from *in vitro* results were used, there were limitations in genotype determination from direct clinical samples. Studies in genotype determination generally multiply the number of tachyzoites through the culture process (Ferreira *et al.*, 2011). This study conducted genotyping directly on the clinical samples of cerebrospinal fluid, showing the need for further investigations. This result also differed from other study in India showing successful application of GRA6 in determining *T. gondii* genotype (Vijaykumar *et al.*, 2016; Vijaykumar *et al.*, 2018). The difference is attributed to the use of postmortem brain specimens from 25 AIDS patients with severe cerebral toxoplasmosis

This study has optimized GRA6 PCR method, based on previous results, by exploring various denaturing, annealing, and extension PCR conditions as well as extending the reaction cycle. Additionally, the nested 2 primers were substituted with an alternative that has shown promising results in initial testing (Gohari *et al.*, 2020). Despite these significant efforts, satisfactory outcomes have not been obtained in our samples.

There have been several markers (multi locus) commonly used to determine *T. gondii* type and genotype. Multilocus PCR-RFLP based on more than six markers is more highly sensitive than using fewer markers. PCR–RFLP genetic markers including SAG1, SAG2, 5’- and 3’-SAG2, alt. SAG2, SAG3, BTUB, GRA6, C22-8, c29-2, L358, PK1, and Apico can be employed for blood, cerebrospinal fluid, and amniotic fluids of patients with cerebral toxoplasmosis and AIDS. (Ferreira *et al.*, 2008; Ferreira *et al.*, 2011)

This study evaluates GRA6 gene as a marker for *Toxoplasma* genotype determination since it is the most widely reported, however it failed to show any positive result on our CSF samples; this is similar to the previously reported studies using ocular fluid.(Kurniawan *et al.*, 2020)

A recent study has significantly explored the molecular epidemiology of *T. gondii*, a parasitic infection, emphasizing the need for continuous studies to refine genotyping methods. In Europe, a similar study was carried out, but the situation still required further investigation due to inconsistent results. A crucial aspect is to enhance the diagnostic accuracy of GRA6 in clinical settings, particularly in complex cases such as Toxoplasmic encephalitis (Fernández-Escobar *et al.*, 2022).

The successful application of GRA6 PCR in control positive sample shows its potential use in certain conditions. However, the difference in results shows the need for a more refined method of GRA6-based genotyping which can be achieved by enhancing the sensitivity of detection methods or integration with other genetic marker to improve diagnostic accuracy in low-parasite-load samples such as CSF (Brenier-Pinchart *et al.*, 2021).

A multi-marker method can also be used to obtain a more comprehensive genotypic profile of *T. gondii* which is aimed at providing a deeper understanding of developing effective strategies to overcome the spread of *T. gondii* (Zhang *et al.*, 2019; Sousa *et al.*, 2023). Future studies should focus on developing sensitive GRA6 detection assays to explore the potential of a multi-marker method in obtaining a more comprehensive genotypic profile of *T. gondii* and avoid propagation (Abedian *et al.*, 2024).

The limitations of this study are caused by several factors contributing to the failure of GRA6 genotyping in CSF samples. The inherently low parasite loads present in CSF pose a significant risk, potentially falling below the detection threshold of GRA6-based PCR assays. Additionally, the unique host-parasite interactions within the central nervous system can affect the expression or detection of GRA6.

## Conclusion

In conclusion, this study showed that GRA6 produced more accurate results in cultured cells, compared to clinical samples obtained directly from HIV/AIDS patients with Toxoplasmic encephalitis. The use of GRA6 has shown promising results, increasing confidence in its effectiveness in determining *T. gondii* genotype. For further investigation, increasing the number of parasites through cell culture proved to be highly beneficial in obtaining better outcomes to identify *T. gondii* genotype.

**Conflict of Interest Statement:** The authors declared that there are no conflicts of interest.

**List of Abbreviations:**
HIV/AIDS: Human Immunodeficiency Virus/Acquired Immune Deficiency Syndrome,
CSF: Cerebrospinal Fluid,
TE: Toxoplasmic encephalitis,
GRA: granule antigen
